# Krüppel-like factor 5 accelerates the pathogenesis of Alzheimer’s disease via BACE1-mediated APP processing

**DOI:** 10.1186/s13195-022-01050-3

**Published:** 2022-07-26

**Authors:** Yaqi Wang, Yuting Cui, Jing Liu, Qiao Song, Min Cao, Yuli Hou, Xiaomin Zhang, Peichang Wang

**Affiliations:** 1 Clinical Laboratory of Xuanwu Hospital, Capital Medical University, Beijing, 100053 People’s Republic of China; 2 Clinical Laboratory of Xuanwu Hospital, National Clinical Research Center for Geriatric Diseases, Beijing, 100053 People’s Republic of China

**Keywords:** Alzheimer’s disease, KLF5, β-Secretase, Amyloid precursor protein

## Abstract

**Background:**

The deposition of β-amyloid (Aβ) in the brain plays a major role in the pathogenesis of Alzheimer’s disease (AD). Aβ is generated via amyloid precursor protein (APP) cleavage through the amyloidogenic pathway. In this pathway, β-secretase (BACE1) is the first and rate-limiting enzyme. Its expression increases through an unknown mechanism in patients with AD. Thus, the key regulatory mechanism of BACE1 in the AD process should be revealed to understand the pathogenesis of AD and explore the key treatment targets of AD.

**Methods:**

Here, APPswe/PS1dE9 (APP/PS1) mice were employed to observe the Krüppel-like factor 5 (KLF5) and BACE1 levels in the serum and brain tissues. HT22 cells were used to explore the relationship between KLF5 and BACE1.

**Results:**

In this study, KLF5 was found to be a novel transcription factor that positively regulated BACE1 by binding to the BACE1 promoter. The KLF5 levels significantly increased not only in the CSF and serum of patients with AD but also in the brain tissue of APP/PS1 mice. They were closely related to cognitive capacity. KLF5 accelerated APP amyloidogenic metabolism and promoted Aβ synthesis through BACE1. Silencing BACE1 could block the KLF5-induced amyloidogenic process of APP. ML264 ameliorated the cognitive deficits and slowed down APP amyloidogenic cleavage in APP/PS1 mice.

**Conclusion:**

The findings above suggest that upregulation of KLF5 might be a critical element in AD progression by accelerating BACE1-mediated APP amyloidogenic cleavage. The inhibition of KLF5 or the combined inhibitory effect of KLF5 and the BACE1 promoter might be a potential strategy to prevent AD pathogenesis.

**Supplementary Information:**

The online version contains supplementary material available at 10.1186/s13195-022-01050-3.

## Background

Alzheimer’s disease (AD), characterized by progressive cognitive decline, is the most common cause of dementia. It ranks fifth among the leading causes of death and affects more than 50 million people worldwide [1]. Although AD has been extensively investigated and many kinds of potential drugs have been tested on animals, drugs that can slow down its progression have yet to be developed. Drug trials on AD treatment have failed likely because of the unknown mechanism of AD, resulting in incorrect treatment targets.

Although the mechanism of AD is complicated, the accumulation of β-amyloid (Aβ) plaques outside neurons and the accumulation of an abnormal form of the protein tau (tau tangles) inside neurons are among the changes that contribute to the damage and destruction of neurons and result in memory loss and other symptoms of AD [2]. As the principal component of AD-associated senile plaques, Aβ is produced via the protease cleavage of the amyloid precursor protein (APP) [3]. In the brain of an individual with AD, APP is cleaved through the amyloidogenic pathway; in this pathway, APP is first cleaved by β-secretase (BACE1) to generate a soluble APPβ (sAPPβ) and a C-terminal fragment protein (CTFβ, also known as C99) [4]. CTFβ is further cleaved by γ-secretase to generate the Aβ peptide of varying chain lengths, including Aβ1-40 and Aβ1-42 [5]. Thus, the regulatory mechanism of BACE1 is likely the key to understanding the pathogenesis of AD [6].

Although BACE1 regulation in AD has been widely explored, its transcriptional regulation has yet to be fully understood. Krüppel-like factor 5 (KLF5, also known as BTEB2) is a C2H2-type zinc finger-containing transcription factor that belongs to the Krüppel-like factor family [7]. It regulates the expression of a wide range of target genes, such as *cyclin D1*, *Egr-1*, *NF-κB*, and *PPARγ* [8–10], so it affects multiple cellular functions, including cell proliferation, differentiation, cell apoptosis, and cell cycle [11, 12]. Although KLF5 is expressed mostly in the epithelia, it is also observed in neuronal cells [13]. Its levels in the AD mouse model also increase [14]. The altered expression of KLF5 target genes is associated with AD pathogenesis [15].

In this study, KLF5 was demonstrated to be a key transcriptional activator of *BACE1*, and the inhibition of the KLF5 expression could ameliorate cognitive deficits and Aβ pathology by reducing APP amyloid cleavage in the AD mouse model.

## Materials and methods

### Human samples

A total of 90 subjects were enrolled for cerebrospinal fluid (CSF) detection: 30 patients with normal cognition including peripheral nerve disease (*n* = 8), myelopathy (*n* = 8), dural arteriovenous fistula (*n* = 5), cerebrovascular malformation (*n* = 4), and body weakness (*n* = 5); 30 patients with mild cognitive impairment (MCI) of Alzheimer’s type; and 30 patients with Alzheimer’s dementia. All the patients were diagnosed by doctors of the Neurology Department of Xuanwu Hospital according to the criteria of NINCDS-ADRDA. Approximately 500 μl of CSF was collected and stored at −80°C for further analyses. Then, 100 subjects were enrolled for serum collection: 30 patients with MCI, 30 patients with Alzheimer’s dementia, and 40 age-matched healthy controls. The same diagnostic criteria were applied. Approximately 1 ml of serum was obtained and stored at −80°C. The design of this study was approved by the ethics committee of Xuanwu Hospital of Capital Medical University and conducted in accordance with the guidelines of the Declaration of Helsinki.

### Animal models

2-, 5-, and 8-month-old male APPswe/PS1dE9 (APP/PS1) mice (*n* = 6 for each age group) and male C57BL/6 mice with the corresponding ages (*n* = 6 for each group) were purchased from Nanjing Biomedical Research Institute of Nanjing University (Nanjing, China). After a week’s balance, the mice were euthanized. Serum samples and brain tissues were collected for further analyses. In a separate experiment, 5-month-old male APP/PS1 mice (*n* = 12) and 5-month-old male C57BL/6 mice (*n* = 6) were used for ML264 administration. APP/PS1 mice were further divided randomly into two groups, namely, the AD control group (*n* = 6) and the ML264-treatment group (*n* = 6). The mice were injected intraperitoneally with 5 mg/kg ML264 (MedChemExpress) or vehicle (PBS) every other day for 15 weeks. After 15 weeks of treatment, the mice were subjected to behavioral tests. At the end of the test, all the animals were sacrificed, and the brain tissues were collected for further analyses. All the mice were housed in a specific pathogen-free room under a 12-h/12-h light/dark cycle and given free access to diet. All the animal procedures were performed in accordance with the criteria outlined in the Guide for the Care and Use of Laboratory Animals (National Institutes of Health, Bethesda, MD) and approved by the Animal Care and Use Committee of Xuanwu Hospital of Capital Medical University.

### Morris water maze test

The Morris water maze is a white circular pool (120 cm in diameter) filled with water and dyed with milk to appear white. The pool was divided into four quadrants of equal areas and labeled clockwise as quadrants A, B, C, and D. A platform was placed at the center of one of the quadrants of the pool and submerged 2 cm below the water. In this test, the platform was set in quadrant B. On the day before the experiment was initiated, the mice were acclimated to swimming for 60 s in the absence of the platform. Then, they were given four times of training with an inter-trial interval of 1 h each day for 4 consecutive days, and their escape latencies were recorded. If a mouse did not reach the platform within 60 s, it would be guided to the platform by the experimenter. On day 5, a probe test was performed. In this test, the platform was removed from the pool with a cutoff time of 60 s, and the time they first crossed the hidden platform and the swimming path was recorded.

### Lentivirus

The full-length KLF5 cDNA (Genbank ID: NM_009769) or APP_swe_ cDNA (Genbank ID: NM_201414) was amplified through polymerase chain reaction (PCR), subsequently cloned into the pLV-CMV vector to construct KLF5-overexpressing lentivirus (KLF5-over) and APP_swe_-overexpressing lentivirus (APP-over), and confirmed by DNA sequencing. Short-hairpin RNA (shRNA) targeting KLF5 (shRNA-KLF5) and BACE1 (shRNA-BACE1) were purchased from Syngentech. The target sequences are shown in Supplementary Table S1. A lentivirus was produced in accordance with the manufacturer’s protocol. The titer of the purified virus was determined through flow cytometry.

### Cell culture and cell treatment

HEK293T, SH-SY5Y, and HT22 cells were purchased from ATCC. HEK293T and HT22 cells were cultured in Dulbecco’s minimum Eagle’s medium (Biological Industries) containing 10% (v/v) fetal bovine serum (Biological Industries) and 1% (v/v) penicillin/streptomycin (Invitrogen, CA, USA), while SH-SY5Y cells were maintained in a minimum essential medium (Biological Industries) supplemented with 10% (v/v) fetal bovine serum (Biological Industries) and 1% (v/v) penicillin/streptomycin at 37°C in a humidified atmosphere containing 5% CO_2_. For lentivirus transfection, HT22 cells were seeded in 12-well culture plates at a cell density of 1 × 10^5^/well 1 day before transfection. Then, lentiviral vectors were transfected into cells by using polybrene. After 72–96 h, the cells were harvested for further analyses. For PMA treatment, PMA (MedChemExpress) was dissolved in DMSO, and SH-SY5Y cells were seeded in 10-cm culture plates at a cell density of 3 × 10^7^/plate 1 day before the treatment. Then, PMA was added to the medium with a final concentration of 100 nM. After 2 h, the cells were harvested for ChIP assay.

### Luciferase assay

For plasmid constructions, the genomic fragments that contained all of the putative KLF5-binding sites on the *BACE1* promoters (~2.0 kb upstream from the transcription start site) and the mutation constructs that mutated the putative KLF5-binding site on the promoter at −1556 to 1547 (site 1), −1467 to −1458 (site 2), −1264 to −1255 (site 3), −988 to −979 (site 4), and −599 to −590 (site 5) were constructed through base synthesis. All the products were subcloned into the *pGL4.1*-promoter vector (Promega, WI, USA) and confirmed by DNA sequencing. For luciferase assay, HEK 293T cells were co-transfected with pcDNA3.1-KLF5 (300 ng) or pcDNA3.1 (300 ng) vector and one of the constructs and pRL-CMV *Renilla* luciferase by using jetOPTIMUS transfection reagent (PolyPlus). After 48 h, the cells were lysed for luciferase activity. Firefly luciferase activity was normalized to *Renilla* luciferase activity.

### RNA extraction and quantitative real-time PCR

Total RNA was extracted from brain tissue samples or cultured cells by using Trizol reagent (Invitrogen) in accordance with the manufacturer’s protocol. A reverse transcription reaction was performed with 1.0 μg of total RNA and 5× All-In-One RT MasterMix (ABM). TB Green^TM^ Premix Ex Taq^TM^ II (TaKaRa) was utilized for quantitative PCR in a final volume of 20 μl on a LightCycler 480 system (Roche) under the following conditions: 95°C for 30 s followed by 40 cycles of amplification (95°C for 5 s and 60°C for 30 s). Melting curve analysis from 60 to 90°C and continuous fluorescence were performed following amplification. The average threshold cycle (Ct) of fluorescence units was applied to analyze the mRNA levels, which were normalized to a housekeeping gene (β-actin or GAPDH). The relative level of each gene was calculated using the 2^-ΔΔCt^ method. The primers are shown in Supplementary Table S2.

### Protein extraction and western blot

The cells or brain tissue samples were homogenized in RIPA buffer to extract total proteins. Protein concentrations were examined using the BCA protein assay (Thermo Fisher Scientific, CA, USA). Equal amounts of total protein were separated on 8% or 15% sodium dodecyl sulfate-polyacrylamide gels and then transferred onto polyvinylidene difluoride membranes (EMD Millipore, Darmstadt, Germany) by using the Trans-Blot Turbo^TM^ blotting system (Bio-Rad). The following primary antibodies were used: rabbit anti-KLF5 (1:1000, Proteintech, 21017-1-AP, Wuhan, China), rabbit anti-BACE1 (1:1000, Abcam, ab183612, Cambridge, UK), and rabbit anti-APP-CTF (1:1000, Proteintech, 27320-1-AP). An enhanced chemiluminescence Western HRP substrate kit (EMD Millipore) was used to detect specific proteins. The target bands were checked in accordance with the “Observed Molecular Weight” of the manufacturer’s instructions (KLF5: 51-55KD, BACE1: 68-70KD) and all target bands were consistent with the reported studies [15, 16].

### Chromatin immunoprecipitation

A chromatin immunoprecipitation (ChIP) assay was performed using a Pierce^TM^ agarose ChIP kit (Thermo Fisher Scientific) in accordance with the manufacturer’s protocol. SH-SY5Y cells and hippocampal tissues of 2-, 5-, and 8-month-old APP/PS1 mice were prepared for the assay. Briefly, the SH-SY5Y cells were treated with PMA (100 nM) for 2 h to induce KLF5, and hippocampal tissues (100–120 mg) were homogenized with a mortar and pestle for single-cell suspension preparation. Then, the cells were cross-linked in 10 ml of 1% formaldehyde (Thermo Fisher), and DNA was fragmented to 200–1000 bp in length through enzyme digestion. Each 150 μl of chromatin solution was immunoprecipitated using 10 μg of monoclonal anti-KLF5 (Santa Cruz, CA, USA) or IgG control overnight at 4°C. Input and immunoprecipitated samples were digested with proteinase K to reverse the cross-linking at 65°C for 90 min. DNA was purified and further analyzed through qPCR by using the primers against the *BACE1* promoter (Supplementary Table S2).

### Immunofluorescence and immunofluorescence laser confocal microscopy assay

Frozen sections of formalin-fixed brain tissues with 5-μm thickness were prepared. They were permeabilized with 0.5% Triton X-100, and antigens were retrieved. Then, the sections were blocked with 3% goat serum and labeled with mouse anti-BACE1 antibody (1:50 dilution; Santa Cruz) and rabbit anti-KLF5 antibody (1:200 dilution; Proteintech) overnight at 4°C. They were subsequently labeled with Texas red-conjugated goat anti-mouse and FITC-conjugated goat anti-rabbit secondary antibodies (Santa Cruz). Nuclei were stained with 4′,6-diamidino-2-phenylindole (Santa Cruz). After being mounted, the samples were analyzed through fluorescence microscopy or laser confocal microscopy.

### Aβ plaque histology

Amyloid plaques were stained with Thioflavin-S. Frozen sections of formalin-fixed brain tissues with 5-μm thickness were prepared, rinsed with TBS thrice, stained with 0.3% Thioflavin-S (Invitrogen) in 50% ethanol for 8 min, washed with 50% ethanol thrice, and rinsed with TBS for 5 min. Then, they were covered with a glass cover by using an antifade mounting medium and observed under a fluorescence microscope.

### β-Secretase activity assay

The activities of BACE1 in cells and mouse brains were determined using a β-secretase activity assay kit (Abcam). Briefly, cells and brain tissues were homogenized with an extraction buffer and then incubated with a reaction buffer and a β-secretase substrate at 37°C for 1 h. The BACE1 activity was measured using a multifunctional plate reader (Beckman Coulter) with Ex/Em = 335/495 nm.

### ELISA

Aβ1-40 and Aβ1-42 levels in the cell culture medium and brain extract and KLF5 levels in the serum were measured using the corresponding sandwich ELISA kits in accordance with the manufacturer’s instructions (Aβ1-40 and Aβ1-42, Mlbio, and KLF5, Abebio, China).

### Statistical analysis

Data were expressed as mean ± SEM and statistically analyzed using two-tailed Student’s test, Mann–Whitney test, one-way ANOVA with Tukey’s test, or two-way ANOVA in GraphPad Prism 6 (GraphPad, La Jolla, CA). Differences among groups were considered to be statistically significant at *P* < 0.05.

## Results

### KLF5 levels in the CSF and serum of patients with AD and AD mouse model increase

**Fig. 1:**
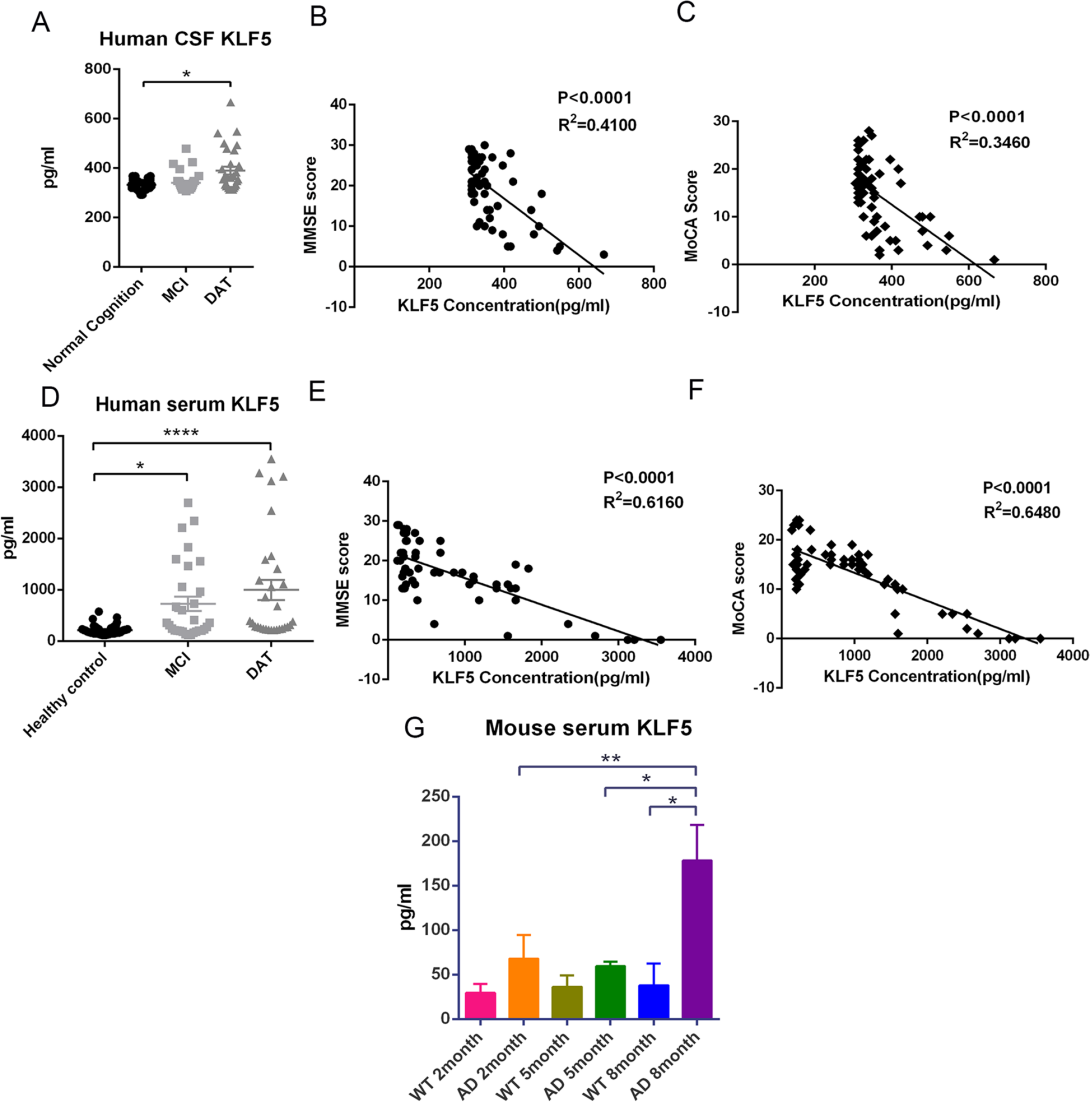
KLF5 levels increase in Alzheimer’s disease (AD) and are associated with cognitive function. **A** KLF5 levels in the CSF of patients with different stages of AD and the normal cognition group (*n* = 30, respectively). **B** Relationship between the KLF concentration in the CSF (*X*-axis) and MMSE scores (*Y*-axis) of patients with AD (*n* = 60). **C** Relationship between the KLF concentration in the CSF (*X*-axis) and MoCA score (*Y*-axis) of patients with AD (*n* = 60). **D** Serum KLF5 levels of patients with different stages of AD (MCI, *n* = 30; DAT, *n* = 30) and the healthy control group (*n* = 40). **E** Relationship of the serum KLF concentration (*X*-axis) and MMSE score (*Y*-axis) of patients with AD (*n* = 60). **F** Relationship between the serum KLF concentration (*X*-axis) and MoCA score (*Y*-axis) of patients with AD (*n* = 60). **G** Serum KLF5 levels of APP/PS1 mice in different months (*n* = 3, respectively). ELISA data in **A**, **D**, and **G** are mean ± s.e.m (*P < 0.05, **P < 0.01, and ****P < 0.0001; one-way ANOVA). Data in **B**, **C**, **E**, and **F** are examined through linear regression analysis

The changes in KLF5 levels were observed initially in patients with AD. Human CSF and serum samples were collected to detect KLF5 levels. The demographic characteristics of the enrolled populations are displayed in Supplementary Tables S3 and S4. In the CSF samples, the KLF5 level slightly increased at the MCI stage, but it was higher in patients with AD–dementia (DAT) than in patients with normal cognition and MCI (Fig. 1A). Then, cognitive scores were analyzed to further explore the relationship between KLF5 and AD progression. The results revealed that the KLF5 level in the CSF was negatively related to the Mini-Mental State Examination (MMSE) and Montreal Cognitive Assessment (MoCA) scores (Fig. 1B, C). In human serum, the KLF5 levels evidently increased at the MCI stage and notably increased at the DAT stage; these levels were 3.37- and 4.61-fold higher than those of the healthy control group, respectively (Fig. 1D). Similarly, serum KLF5 levels were strongly correlated with MMSE/MoCA scores (Fig. 1E, F). The analysis of the KLF5 level in the serum of APP/PS1 mice at different ages revealed that the serum KLF5 of 8-month-old APP/PS1 mice increased compared with that of 2- and 5-month-old APP/PS1 mice and 8-month-old WT mice (Fig. 1G). These results indicated that KLF5 expression increased with AD progression and might play a potential role in this process.

### KLF5 and BACE1 are upregulated in the brain tissues of APP/PS1 mice

**Fig. 2:**
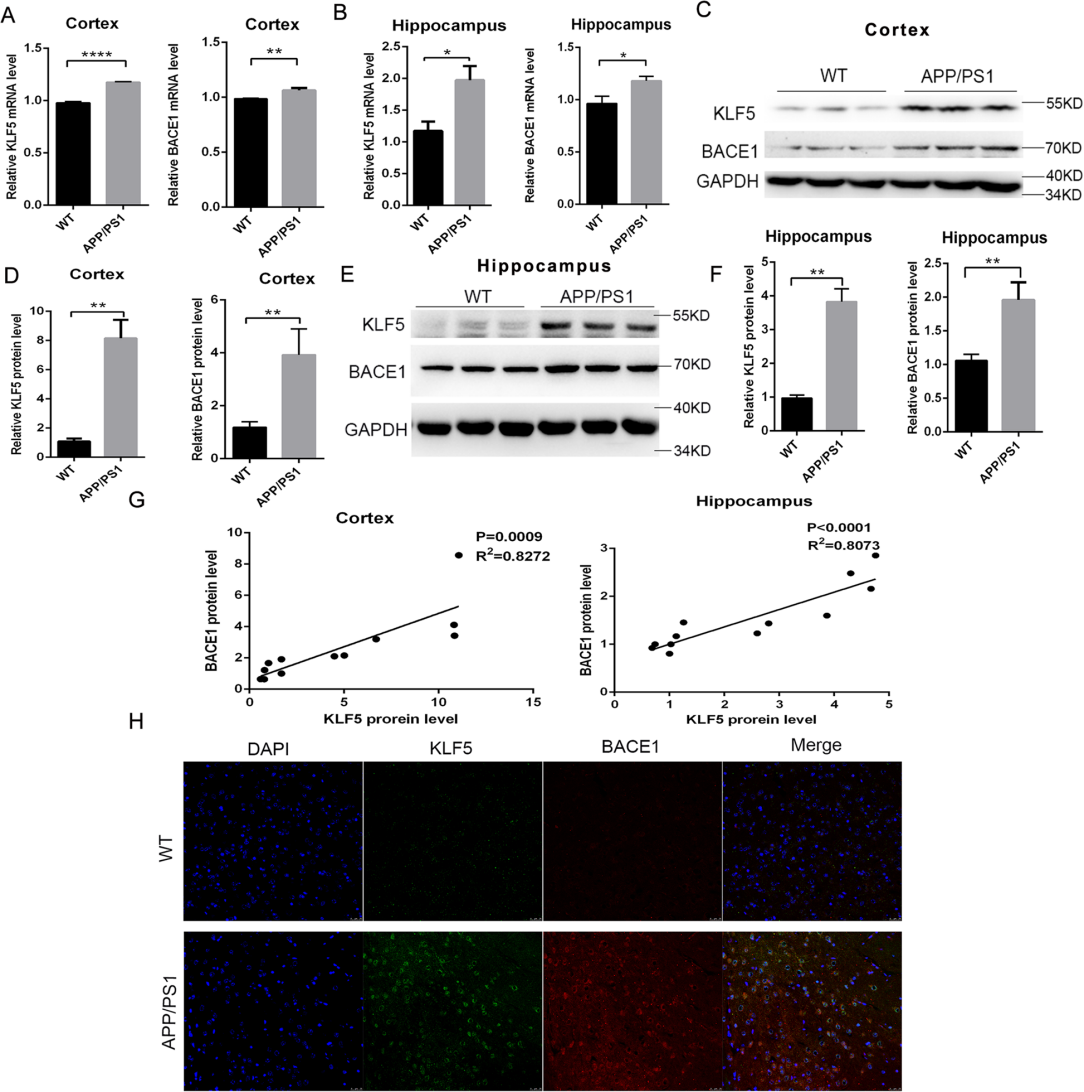
KLF5 and BACE1 expression levels increase in APP/PS1 mice and are associated with cognitive function. **A** mRNA levels of KLF5 and BACE1 in the cerebral cortex of APP/PS1 mice (*n* = 6) and WT mice (*n* = 6). **B** mRNA levels of KLF5 and BACE1 in the hippocampus of APP/PS1 mice (*n* = 6) and WT mice (*n* = 6). **C** Representative Western blots of the protein expression levels of KLF5 (upper panel) and BACE1 (middle panel) in the cerebral cortex of APP/PS1 mice and WT mice. **D** Relative quantification of the protein levels of KLF5 and BACE1 in the cerebral cortex of APP/PS1 mice (*n* = 6) and WT mice (*n* = 6). **E** Representative Western blots of the protein expression levels of KLF5 (upper panel) and BACE1 (middle panel) in the hippocampus of APP/PS1 mice and WT mice. **F** Relative quantification of the protein levels of KLF5 and BACE1 in the hippocampus of APP/PS1 mice (*n* = 6) and WT mice (*n* = 6). **G** Relationship between the protein levels of KLF5 (*X*-axis) and BACE1 (*Y*-axis) in the brain of the mice (*n* = 12). **H** Immunofluorescence laser confocal microscopy of the expression and sublocalization of KLF5 and BACE1 in the brain of APP/PS1 mice and WT mice. Data in **A**, **B**, **D**, and **F** are mean ± s.e.m (*P < 0.05, **P < 0.01, ****P < 0.0001; *t*-test). Data in **G** are examined through linear regression analysis

The KLF5 level in the brain tissues of APP/PS1 mice was then determined. The KLF5 and BACE1 transcript levels in the cortex and hippocampal tissues of 8-month-old APP/PS1 mice increased significantly (Fig. 2A, B). The protein levels of KLF5 and BACE1 were also strongly upregulated in the brain of 8-month-old APP/PS1 mice (Fig. 2C–F). KLF5 levels were strongly correlated with BACE1 levels (Fig. 2G). Immunocytochemistry confocal assay showed that the expression levels of KLF5 and BACE1 in 8-month-old APP/PS1 mice were higher, and KLF5 was co-located with BACE1 in the cytoplasm of neurons (Fig. 2H). These data also indicated that KLF5 could be associated closely with the BACE1 expression in AD.

### KLF5 positively regulates the BACE1 expression, accelerates APP amyloidogenic metabolism, and promotes Aβ synthesis

**Fig. 3:**
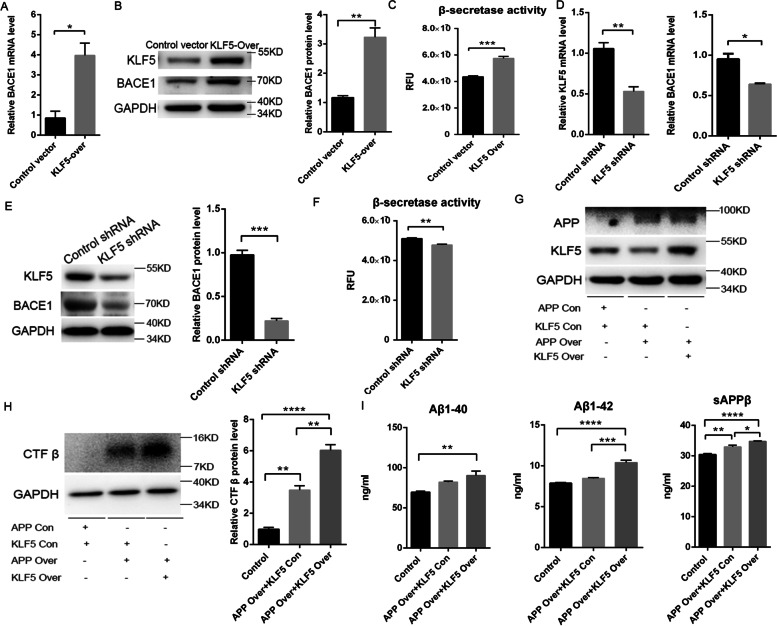
KLF5 regulates the BACE1 expression and promotes amyloidogenic APP processing in HT22 cells. **A** mRNA level of BACE1 in HT22 cells transfected with KLF5 or the control vector. **B** Representative Western blots and relative quantification of the protein expression levels of KLF5 and BACE1 in HT22 cells transfected with KLF5 or the control vector. **C** BACE1 activity (fluorogenic BACE1 activity assay) in HT22 cells transfected with KLF5 or the control vector. **D** mRNA levels of KLF5 and BACE1 in HT22 cells transfected with KLF5 shRNA or control-shRNA. **E** Representative Western blots and relative quantification of the protein expression levels of KLF5 and BACE1 in HT22 cells transfected with KLF5 shRNA or control-shRNA. **F** BACE1 activity (fluorogenic BACE1 activity assay) in HT22 cells transfected with KLF5 shRNA or control-shRNA. **G** Representative Western blots of the protein expression levels of APP (upper panel) and KLF5 (middle panel) in HT22 cells co-transfected with APP_swe_ and KLF5 or the control vector. **H** Representative Western blots and relative quantification of the protein expression of APP-CTFβ in HT22 cells co-transfected with APP_swe_ and KLF5 or the control vector. **I** Levels of Aβ40, Aβ42, and sAPPβ in the medium of HT22 cells co-transfected with APP_swe_ and KLF5 or the control vector. Data are mean ± s.e.m of three separate experiments (*P < 0.05, **P < 0.01, ***P < 0.001, and ****P < 0.0001; *t*-test with parametric data, Mann–Whitney test with nonparametric data, and one-way ANOVA)

The KLF5 levels increased in the patients and mice with AD, and this increase was associated with BACE1 expression. As such, our hypothesis was that KLF5 would upregulate the BACE1 transcription and protein levels, resulting in Aβ pathology. The BACE1 level was analyzed in mouse neuronal HT22 cells with ectopic expression or KLF5 knockdown. The mRNA and protein levels of BACE1 were enhanced in the cells with KLF5 overexpression (Fig. 3A, B). The KLF5 overexpression also induced the β-secretase activity (Fig. 3C). On the contrary, KLF5 knockdown reduced the mRNA and protein expression levels of BACE1 (Fig. 3D, E). The β-secretase activity also decreased significantly (Fig. 3F). These results indicated that KLF5 positively regulated the BACE1 expression.

APP is initially cleaved by BACE1 in AD. BACE1 cleavage induces the production of C-terminal APP-CTFβ and N-terminal sAPPβ. In this study, HT22 cells were co-transfected with KLF5 and APP_swe_ to investigate the effect of KLF5 on APP metabolism (Fig. 3G). The results showed that APP-CTFβ and sAPPβ in the cells co-transfected with APP_swe_ and KLF5 were notably induced, thereby significantly inducing Aβ40 and Aβ42 compared with those that only overexpressed APP_swe_ (Fig. 3H, I). Therefore, KLF5 likely accelerated the APP amyloidogenic pathway and promoted Aβ42 synthesis.

### KLF5 binds to BACE1 promoter to activate BACE1 transcription

**Fig. 4:**
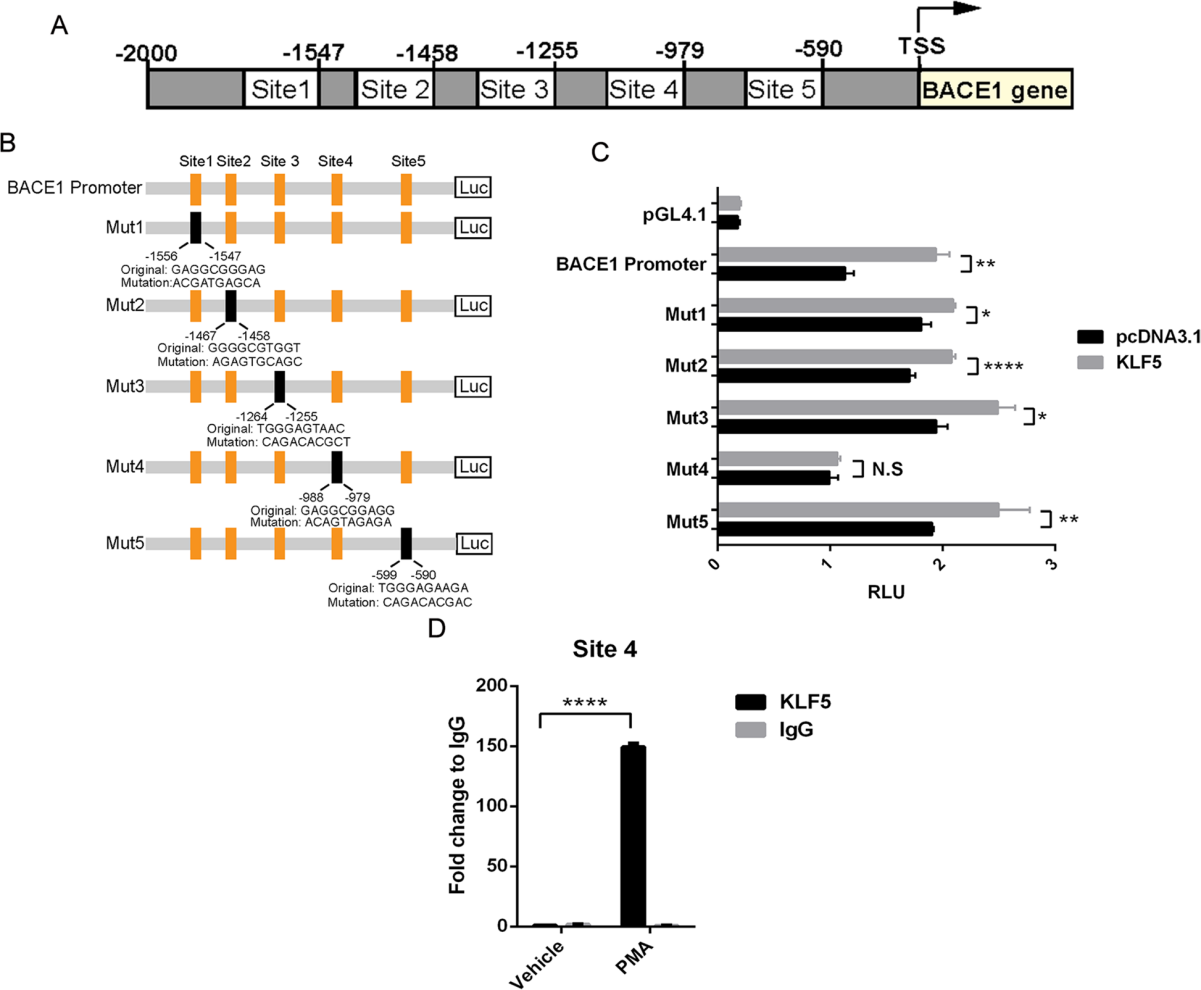
KLF5 binds to the BACE1 promoter and regulates BACE1 transcription. **A** Putative KLF5 binding sites on human BACE1 promoter predicted by the JASPAR database. **B** Schematic of the mutation constructs used to identify the KLF5-binding site on the BACE1 promoter. The mutated sequences are shown. **C** Promoter activities determined by luciferase assay in HEK293 cells transfected with KLF5 or pcDNA3.1 and human BACE1 promoter luciferase reporter plasmid (−2000 to TSS) or different mutations. **D** ChIP-qPCR assay of site 4 in SH-SY5Y cells treated with PMA or vehicle for 2 h. Data are mean ± s.e.m of three separate experiments (*P < 0.05, **P < 0.01, and ****P < 0.0001; *t*-test)

Previous studies showed that KLF5 binds to a GC-rich sequence of its target genes [7, 17]. In the present study, the role of KLF5 in the transcription of BACE1 was analyzed to investigate the mechanism by which KLF5 positively regulated BACE1 expression. Sequence analysis was performed on the JASPAR database, and the five putative KLF5-binding sites, located upstream from the transcriptional start site, were predicted within the human *BACE1* promoter (Fig. 4A). The predicted results indicated that the putative KLF5-binding sites could be located within −599 to −590 (site 5), −988 to −979 (site 4), −1264 to −1255 (site 3), −1467 to −1458 (site 2), and −1556 to −1547 (site 1). The point mutants of the five putative KLF5-binding sites and the overall length of the *BACE1* promoter were synthesized to construct the *BACE1* promoter-driven reporters (Fig. 4B). Each of these fragments and the control reporter (basic pGL4.1 empty vector) were co-transfected with KLF5 or the control vector in HEK293T cells, and the luciferase activity was detected. The results showed that the luciferase activity of BACE1 promoter was significantly enhanced in KLF5-overexpressing cells compared with that in the cells transfected with control vector, suggesting that KLF5 promoted the activity of the *BACE1* promoter. When site 4 was mutated, KLF5 could hardly activate *BACE1* promoter transcription, whereas other KLF5-binding site mutants still significantly responded to the KLF5 overexpression (Fig. 4C). The ChIP assay was performed to identify whether site 4 was the actual binding site of KLF5 in the *BACE1* promoter. The KLF5 expression is rapidly induced in NIH3T3 and LNCaP cells exposed to phorbol 12-myristate 13-acetate (PMA), which promotes the BACE1 expression in U937 cells [18–20]. Thus, SH-SY5Y cells, which are commonly used in studying AD, were stimulated with PMA in our study to investigate whether KLF5 and BACE1 expression could be induced by PMA stimulation. Our results showed that PMA induced both KLF5 and BACE1 in SH-SY5Y cells (Supplementary Fig. S1). Sequentially, the ChIP assay was conducted using an anti-KLF5 antibody and the primers flanking the KLF5-binding site on the *BACE1* promoter. The results revealed that the KLF5 binding to site 4 on the *BACE1* promoter significantly increased in the PMA-treated SH-SY5Y cells compared with that in the controls (Fig. 4D). Therefore, site 4 was a major KLF5-binding site on the *BACE1* promoter.

Furthermore, the ChIP assay was conducted by using hippocampus tissues of APP/PS1 mice of different ages to investigate whether the binding level of KLF5 with the *BACE1* promoter changed in AD. Three putative KLF5-binding sites are present in the proximal promoter regions of the mouse *BACE1* promoter predicted by the JASPAR database. They were located within −1956 to −1947 (site 1), −1938 to −1929 (site 2), and −526 to −517 (site 3; Supplementary Fig. S2A). Our ChIP results verified that KLF5 bound to all putative sites in the cortex of 8-month-old mice, and the binding level dramatically increased in APP/PS1 mice compared with that in WT mice (Supplementary Fig. S2B). Moreover, the KLF5-binding level gradually increased in the hippocampal tissues with the aging of APP/PS1 mice (Supplementary Figs. S2C, D). These results revealed that KLF5 was an important transcriptional factor that promoted APP amyloidogenic cleavage by upregulating the BACE1 expression in AD progression.

### Silencing BACE1 blocks the KLF5-induced amyloidogenic processing of APP

**Fig. 5:**
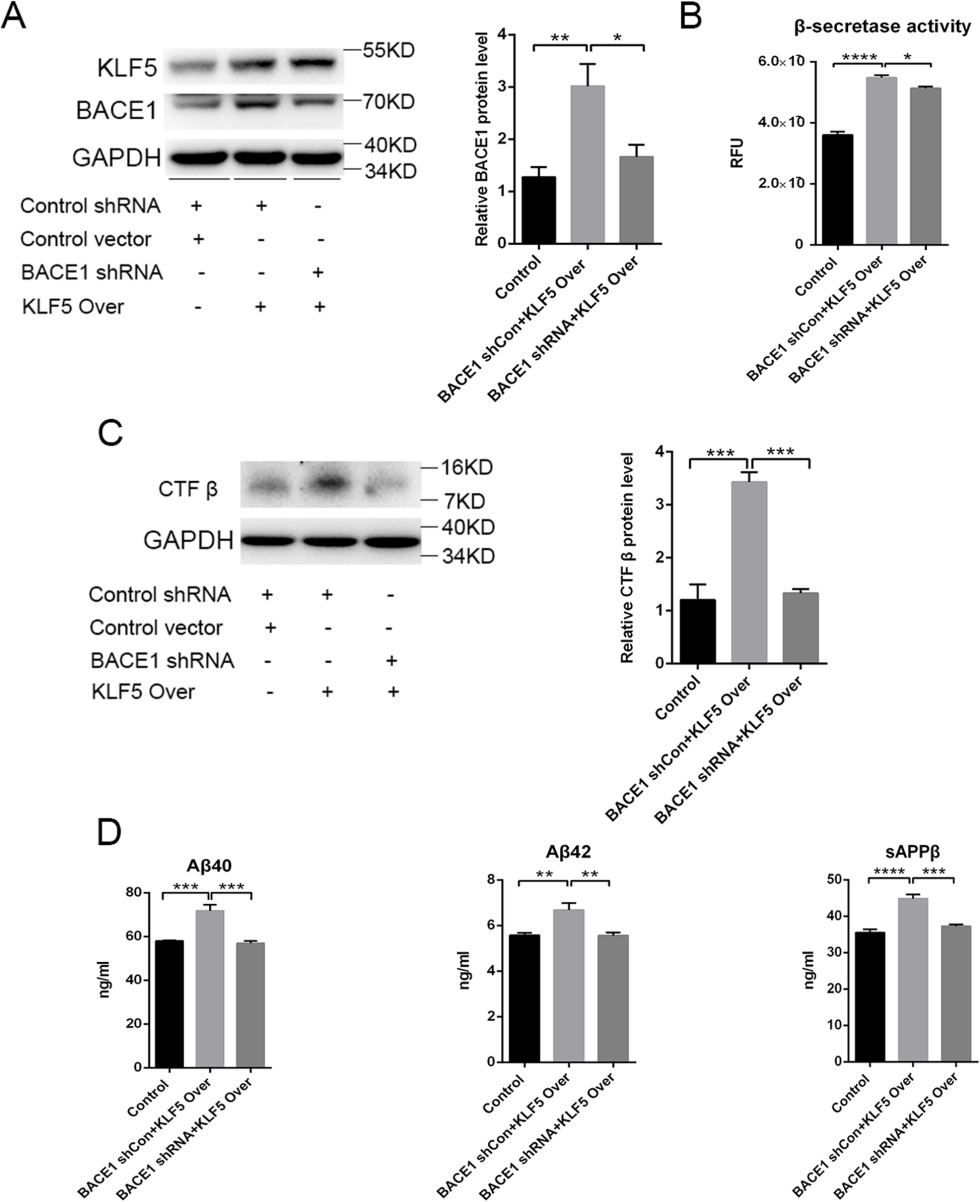
Knockdown of BACE1 blocks KLF5-induced amyloidogenic APP processing in HT22 cells. **A** Representative Western blots and relative quantification of BACE1 protein in HT22 cells co-transfected with BACE1-shRNA or control-shRNA and KLF5 or the control vector. **B** BACE1 activity (fluorogenic BACE1 activity assay) in HT22 cells co-transfected with BACE1-shRNA or control-shRNA and KLF5 or the control vector. **C** Representative Western blots and relative quantification of APP-CTFβ protein in HT22 cells co-transfected with BACE1-shRNA or control-shRNA and KLF5 or the control vector. **D** Levels of Aβ40, Aβ42, and sAPPβ in the medium of HT22 cells co-transfected with BACE1-shRNA or control-shRNA and KLF5 or control vector. Data are mean ± s.e.m of three separate experiments (*P < 0.05, **P < 0.01, ***P < 0.001, and ****P < 0.0001; one-way ANOVA)

BACE1 in HT22 cells was silenced using BACE1-shRNA to further demonstrate whether KLF5 promoted APP metabolism and Aβ synthesis by activating BACE1. The cells were divided into three groups: the control group, the KLF5 overexpression group, and the BACE1 knockdown group. The cells in these groups were co-transfected with control-shRNA and control vector, control-shRNA and KLF5-over vector, and BACE1-shRNA and KLF5-over vector, respectively. Our results showed that KLF5 overexpression notably upregulated the protein expression of BACE1 and enhanced β-secretase activity, whereas silencing BACE1 evidently decreased the protein expression of BACE1 and β-secretase activity (Fig. 5A, B). Remarkably, the APP-CTFβ and sAPPβ levels were induced by KLF5 overexpression, and the Aβ40 and Aβ42 levels significantly increased. These results suggested an accelerated process of APP amyloidogenic cleavage. However, the levels of APP-CTFβ, sAPPβ, Aβ40, and Aβ42 were downregulated in the cells co-transfected with BACE1-shRNA and KLF5-overexpression vector compared with those in the cells transfected only with BACE1-shRNA or KLF5-overexpression vector (Fig. 5C, D). This finding suggested that the promoted effect of APP amyloidogenic cleavage induced by KLF5 was nearly blocked when BACE1 was silenced. Therefore, KLF5-induced amyloidogenic APP processing and Aβ production were attributed to the BACE1 expression mediated by KLF5.

### ML264 ameliorates cognitive deficits and Aβ deposition by reducing the BACE1 expression in APP/PS1 mice

**Fig. 6:**
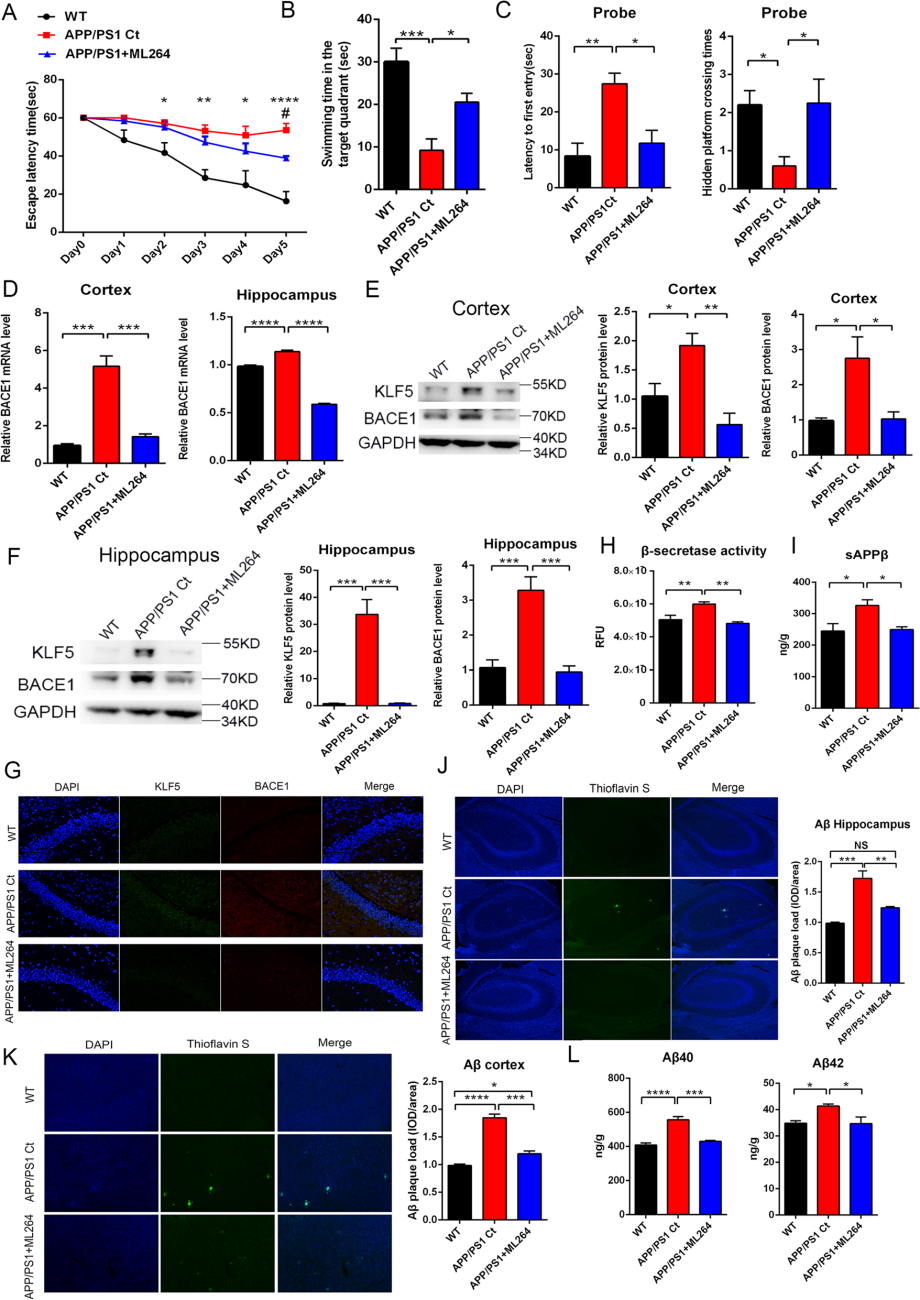
Administration of ML264 inhibits the BACE1 expression and ameliorates memory impairments in APP/PS1 mice. Five-month-old APP/PS1 mice were treated with vehicle (PBS) or ML264 (5 mg/kg, i.p, every other day) for 15 weeks. **A**–**C** WT mice (WT, *n* = 6), vehicle-treated APP/PS1 mice (APP/PS1 Ct, *n* = 6), and ML264-treated APP/PS1 mice (APP/PS1+ML264, *n* = 6) were trained and tested on the spatial memory version of the Morris water maze. **A** The escape latency time of the different groups of mice was recorded on each training day (means ± s.e.m; one-way ANOVA; *: WT versus APP/PS1 Ct, *P < 0.05, **P < 0.01, and ****P < 0.0001; #: APP/PS1 Ct versus APP/PS1+ML264, ^#^*P* < 0.05). **B** Mice were given a memory retention probe trial with the escape platform removed 24 h after the last training trial. The swimming time in the target quadrant of the different groups of mice was recorded. **C** Time for the first crossing over the original platform site and the platform crossing time of different groups of mice. **D** mRNA level of BACE1 in the cerebral cortex (left) and hippocampus (right) of different groups of mice. **E** Representative Western blots and relative quantification of the protein expression levels of KLF5 and BACE1 in the cerebral cortex of different groups of mice. **F** Representative Western blots and relative quantification of the protein expression levels of KLF5 and BACE1 in the hippocampus of different groups of mice. **G** Immunofluorescence (×200) of the expression levels of KLF5 and BACE1 in the brain of different groups of mice. **H** BACE1 activity (fluorogenic BACE1 activity assay) in the brain of different groups of mice. **I** Levels of sAPPβ in the brain of different groups of mice. **J** Staining and quantification of amyloid plaques in the hippocampus among different groups of mice. **K** Staining and quantification of amyloid plaques in the cortex among different groups of mice. **L** Levels of Aβ40 and Aβ42 in the brain of different groups of mice. Data in **B**–**F**, **H**, **I**, **J**, **K**, and **L** are mean ± s.e.m (*P < 0.05, **P < 0.01, ***P < 0.001, and ****P < 0.0001; one-way ANOVA)

Five-month-old APP/PS1 mice were treated with 5 mg/kg body weight ML264 to investigate whether KLF5 inhibition could improve the cognitive capacity by reducing the BACE1 expression in vivo every other day for 15 weeks. Five-month C57/BL6 WT mice and 5-month APP/PS1 mice administrated with vehicle (PBS) were as WT mice control and mice with AD control. At the end of the time point, a Morris water maze test was performed to detect cognitive function. The results of the latency to the hidden platform showed that the control mice with AD spent longer time from days 2 to 5 than the WT control mice. This result validated the significant impairment of learning and memory in AD control mice. By contrast, the mice treated with ML264 displayed an evident shorter escape latency in finding the hidden platform than that of AD control mice after training (Fig. 6A). During the probe trial in the hidden platform task, WT and ML264-treated mice spent more time swimming in the target quadrant than the AD control mice (Fig. 6B) and spent significantly less time swimming in the other quadrants (Supplementary Fig. S3). Besides, both WT and ML264-treated mice spent less time first to cross the original location of the platform and crossed obviously more times to reach the target platform compared to AD mice (Fig. 6C). These data showed that ML264 strongly improved the impaired learning and memory of APP/PS1 mice.

The BACE1 expression, APP cleavage, and Aβ synthesis were examined among the groups. The results showed that the mRNA and protein levels of BACE1 were upregulated in the brain tissues of the AD control mice compared with that in the WT mice but dramatically downregulated in the ML264-treated mice (Fig. 6D–G). The β-secretase activity level was consistent with the BACE1 expression in all groups of mice (Fig. 6H). Likewise, the sAPPβ, Aβ40, and Aβ42 levels decreased significantly in the brain tissue of ML264-treated mice compared with those in the AD control mice (Fig. 6I, L). The Aβ plaques were obviously reduced in the brain of ML264-treated mice (Fig. 6J, K). These data demonstrated that the inhibition of KLF5 efficiently ameliorated cognitive deficits and Aβ accumulation by downregulating the BACE1 expression.

## Discussion

Previous studies showed that KLF5 participates in several diseases associated with the central nervous system (CNS). It is a novel susceptibility gene for schizophrenia, and its expression is involved in the pathophysiology of schizophrenia via glutamatergic neurotransmission [13]. The inhibition of the KLF5-mediated JNK signaling pathway efficiently attenuates neuronal apoptosis in rats with ischemic stroke [21]. Moreover, the KLF5-NF-κB signaling pathway is activated in mice with AD, and the inhibition of this pathway improves the cognitive function of mice with AD [14], which reveal that KLF5 indeed plays an important role in AD. In our study, we showed that KLF5 levels were highly expressed in patients with AD and mice with AD and strongly associated with memory and learning function (Figs. 1 and 2). Therefore, KLF5 was involved in AD pathogenesis.

Aβ aggregation in the brain triggers a series of neurotoxic effects, resulting in neuronal dysfunction, inflammation, and neuronal loss [22–24], which eventually lead to the occurrence of AD. BACE1 is the key enzyme in Aβ production, and the mRNA and protein levels of BACE1 are upregulated in AD [25–27]. A high BACE1 expression is one of the key factors of Aβ accumulation in AD. However, the regulatory mechanism of the BACE1 expression remains poorly understood. In this study, changes in KLF5 levels were associated with BACE1 levels in AD progression, and the KLF5 overexpression induced the BACE1 expression. These results suggested that KLF5 positively regulated the BACE1 expression in AD. KLF5 also affected APP metabolism. APP is cleaved by BACE1, which produces APP-CTFβ and sAPPβ in AD. Our results showed that the levels of APP-CTFβ and sAPPβ were significantly increased in cells with KLF5 overexpression, indicating that KLF5 promoted APP cleavage toward the amyloidogenic pathway; thus, it accelerated Aβ generation (Fig. 3). Moreover, when BACE1 was silenced in neuronal cells, the KLF5-mediated increase in Aβ production was nearly inhibited (Fig. 5). Therefore, KLF5 possibly induced Aβ synthesis by activating BACE1.

BACE1 transcription is regulated by multiple transcription factors as activators or repressors, including SP1, YY1, EGR1, NRF2, NF-κB, PPAR-γ, and HIF1. SP1 and YY1 bind to the *BACE1* promoter and activate the BACE1 expression in PC12 cells [28, 29]. EGR1 is also a transcriptional activator of BACE1 in the brain of individuals with AD [15]. By contrast, NRF2 likely binds to the ARE element of the *BACE1* promoter and negatively regulates the BACE1 expression [30]. NF-κB exhibits a unique cell type-specific regulation of BACE1 promoter activity [31, 32]. It activates BACE1 transcription in activated astrocytic and Aβ-exposed neuronal cells while suppressing BACE1 expression in differentiated neuronal cells and nonactivated glial cells. In addition, PPAR-γ inhibits the BACE1 expression by antagonizing the activities of other transcription factors [33, 34], and HIF1 binds to the *BACE1* promoter only under hypoxic conditions [35]. In our study, KLF5 bound to the *BACE1* gene promoter, thus promoting the BACE1 expression (Fig. 4). Furthermore, the level of KLF5 binding to the *BACE1* gene promoter increased with aging in mice with AD (Supplementary Fig. S2). These data revealed that KLF5 was involved in AD pathogenesis by acting as a transcription factor directly targeting BACE1. Therefore, the mechanism of regulating BACE1 expression was a complex network.

Cognitive deficits and Aβ deposition are alleviated in mice with AD models by reducing BACE1. The NRF2 inducer sulforaphane ameliorates AD-related cognitive deficits by downregulating *BACE1* and *BACE1-AS* expression levels and subsequently inducing the Aβ generation in both 5×FAD and 3×Tg-AD mice [29]. Conversely, progranulin administration attenuates Aβ deposition in the hippocampus of 5×FAD mice by modulating the BACE1 expression [36]. ML264 is a novel small molecular compound that selectively inhibits the KLF5 expression [37]. ML264 suppresses the growth and metastasis in colorectaloma and osteosarcoma [37, 38]. In our research, ML264, a KLF5 inhibitor, promoted the cognitive function of mice with AD and reduced the accumulation of Aβ in the brain of APP/PS1 mice by suppressing the BACE1 expression (Fig. 6). This finding strongly supported the key role of KLF5 in the AD process. Given the recent failures of clinical trials involving the enzymatic inhibitors of BACE1 to treat AD [39–41], downregulating the BACE1 expression via KLF5 inhibition or blocking the binding of KLF5 to BACE1 promoter might be alternative approaches.

## Conclusion

**Fig. 7:**
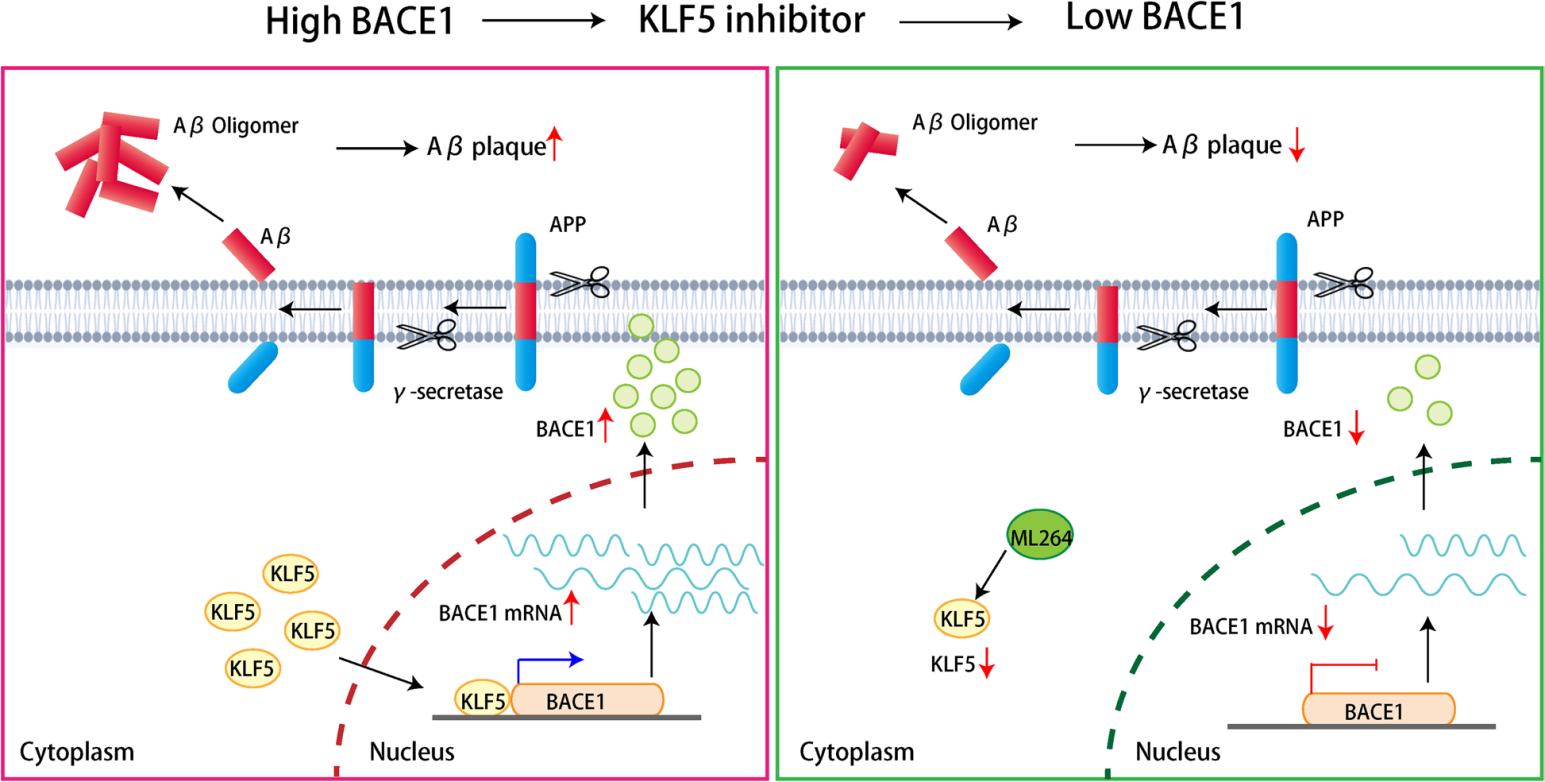
Diagram showing the mechanism by which KLF5 positively regulates BACE1 expression. KLF5 directly binds to the BACE1 promoter and activates its transcription

Overall, KLF5 participates in AD pathogenesis by activating the expression of BACE1 as a transcription factor (Fig. 7). The repression of KLF5 ameliorates cognitive deficits and protects against Aβ deposition in APP/PS1 mice by downregulating BACE1. Our study proposes a potential strategy for AD modification through the inhibition of KLF5.

## Supplementary Information

**Additional file 1: Supplementary Figure S1.** KLF5 and BACE1 act in response to PMA exposure in SH-SY5Y cells. A Time course of KLF5 expression during PMA exposure. B Time course of the BACE1 expression during PMA exposure. Real-time PCR of cells treated with PMA for each indicated time point. Data are mean ± s.e.m of three separate experiments (*P < 0.05 and ****P < 0.0001; two-way ANOVA).

**Additional file 2: Supplementary Figure S2.** Binding level of KLF5 to BACE1 promoter increases with aging in the brain tissues of APP/PS1 mice. A Putative KLF5 binding sites on mouse BACE1 promoter predicted by the JASPAR database. B ChIP-qPCR assay of putative KLF5 binding sites in the cortex of 8-month-old WT and APP/PS1 mice (*n* = 3, respectively). C ChIP-qPCR assay of Site 1/2 in the hippocampal tissues of 2-, 5-, and 8-month-old APP/PS1 mice. D ChIP-qPCR assay of Site 3 in the hippocampal tissues of 2-, 5-, and 8-month-old APP/PS1 mice (*n* = 3, respectively). Data are mean ± s.e.m (**P < 0.01 and ****P < 0.0001; one-way ANOVA).

**Additional file 3: Supplementary Figure S3.** The swimming time in each quadrant of the different groups of mice. A The swimming time in each quadrant of WT mice (*n* = 6). B The swimming time in each quadrant of vehicle-treated APP/PS1 mice (*n* = 6). C The swimming time in each quadrant of ML264-treated APP/PS1 mice (*n* = 6). Data in A-C are mean ± s.e.m (*P < 0.05, **P < 0.01, and ***P < 0.001; one-way ANOVA).

**Additional file 4: Supplementary Table S1.** Target sequences of short-hairpin RNA (shRNA) targeting KLF5 (KLF5-shRNA) and BACE1 (BACE1-shRNA).

**Additional file 5: Supplementary Table S2.** Primer sequences for real-time PCR and ChIP-qPCR.

**Additional file 6: Supplementary Table S3.** Characterization of patients in different CSF groups. MCI: Mild cognitive impairment; DAT: Alzheimer’s type of dementia; MMSE: Mini-Mental State Examination; and MoCA: Montreal Cognitive Assessment (****P < 0.0001; data versus MCI; Student’s test). MCI: Mild cognitive impairment; DAT: Alzheimer’s type of dementia. ****P<0.0001, the data were analyzed by Student’s test, vs. MCI.

**Additional file 7: Supplementary Table S4.** Characterization of patients in different serum groups. HC: Healthy control; MCI: Mild cognitive impairment; and DAT: Alzheimer’s type of dementia (*P < 0.05; data versus MCI; Student’s test). HC: Healthy control; MCI: Mild cognitive impairment; DAT: Alzheimer’s type of dementia. *P<0.05, the data were analyzed by Student’s test, vs. MCI.

## Data Availability

The data in this study have been deposited to the Zenodo (DOI: 10.5281/zenodo.4784947).

## Abbreviations

Aβ: β-Amyloid
AD: Alzheimer’s disease
APP: Amyloid precursor protein
BACE1: β-Site APP cleaving enzyme 1
ChIP: Chromatin immunoprecipitation
CTFβ: C-terminal fragment beta
GAPDH: Glyceraldehyde-3-phosphate dehydrogenase
HRP: Horseradish peroxidase
KLF5: Krüppel-like factor 5
MMSE: Mini-Mental State Examination
MoCA: Montreal Cognitive Assessment
PMA: Phorbol ester
PS1: Presenilin-1
sAPPβ: Soluble amyloid precursor protein beta
ShRNA: Short-hairpin RNA
