# Supplementary figures and images for: Krüppel-like factor 5 accelerates the pathogenesis of Alzheimer’s disease via BACE1-mediated APP processing
Source: Alzheimers Res Ther. 2022 Jul 26;14:103. doi: 10.1186/s13195-022-01050-3 (PMC9316766; doi:10.1186/s13195-022-01050-3)

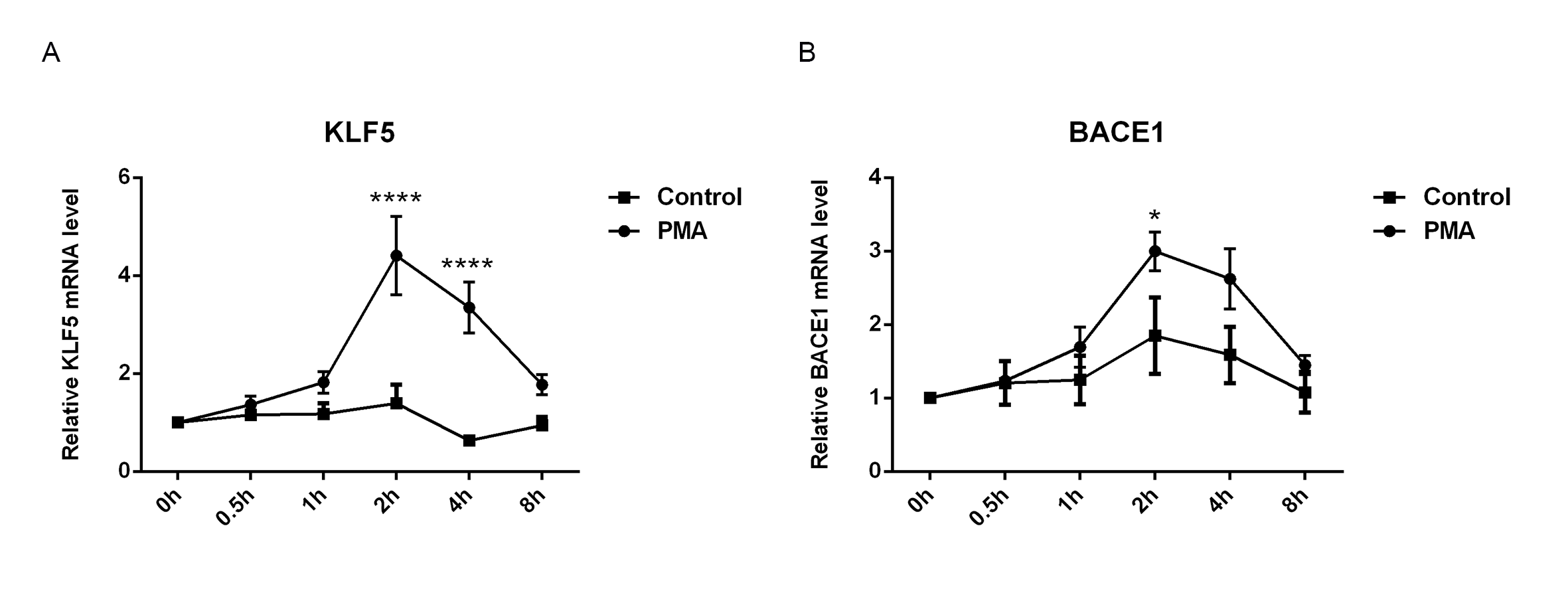

Supplement: Supplementary file 1 — Additional file 1: Supplementary Figure S1. KLF5 and BACE1 act in response to PMA exposure in SH-SY5Y cells. A Time course of KLF5 expression during PMA exposure. B Time course of the BACE1 expression during PMA exposure. Real-time PCR of cells treated with PMA for each indicated time point. Data are mean ± s.e.m of three separate experiments (*P < 0.05 and ****P < 0.0001; two-way ANOVA). [file 13195_2022_1050_MOESM1_ESM.tif]

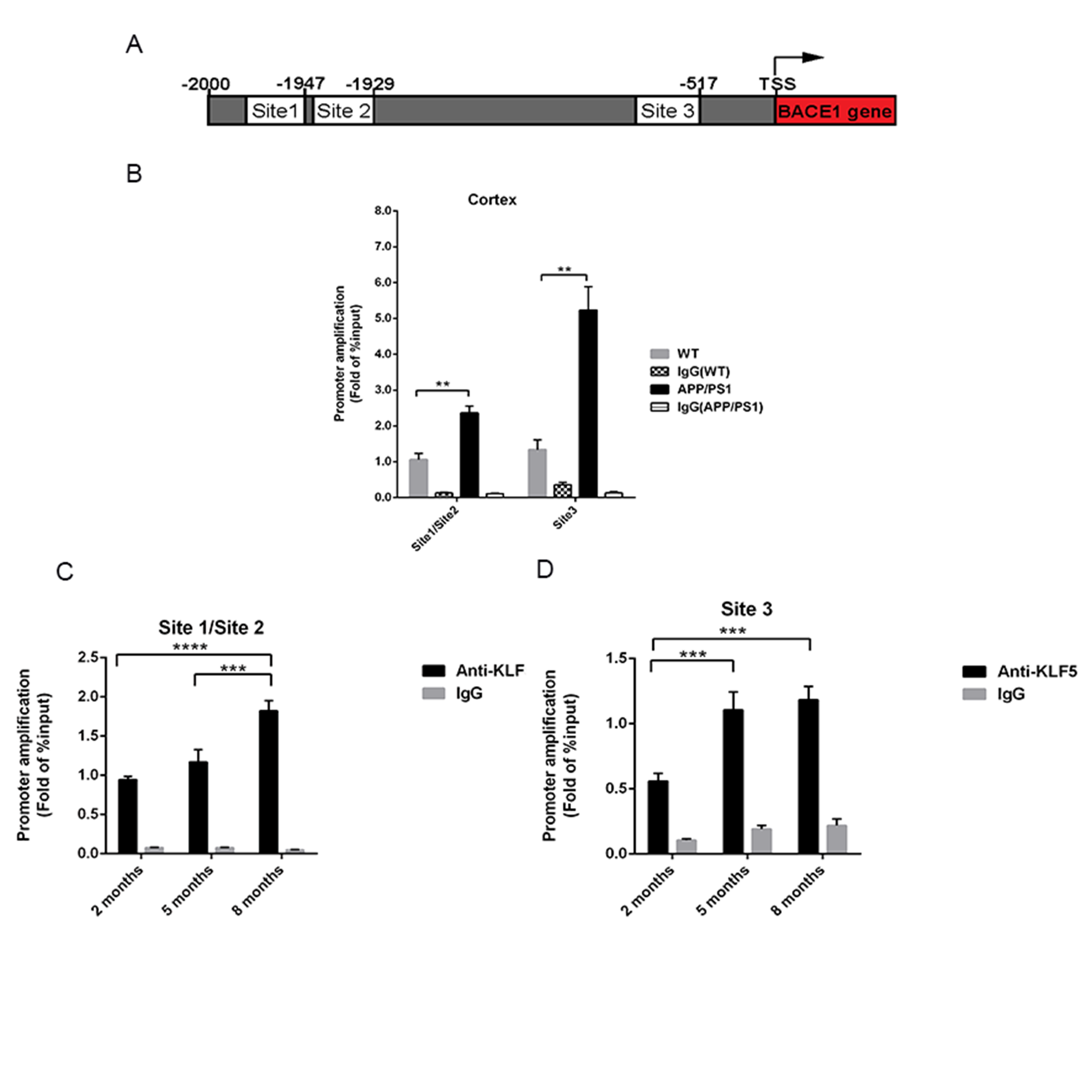

Supplement: Supplementary file 2 — Additional file 2: Supplementary Figure S2. Binding level of KLF5 to BACE1 promoter increases with aging in the brain tissues of APP/PS1 mice. A Putative KLF5 binding sites on mouse BACE1 promoter predicted by the JASPAR database. B ChIP-qPCR assay of putative KLF5 binding sites in the cortex of 8-month-old WT and APP/PS1 mice (n = 3, respectively). C ChIP-qPCR assay of Site 1/2 in the hippocampal tissues of 2-, 5-, and 8-month-old APP/PS1 mice. D ChIP-qPCR assay of Site 3 in the hippocampal tissues of 2-, 5-, and 8-month-old APP/PS1 mice (n = 3, respectively). Data are mean ± s.e.m (**P < 0.01 and ****P < 0.0001; one-way ANOVA). [file 13195_2022_1050_MOESM2_ESM.tif]

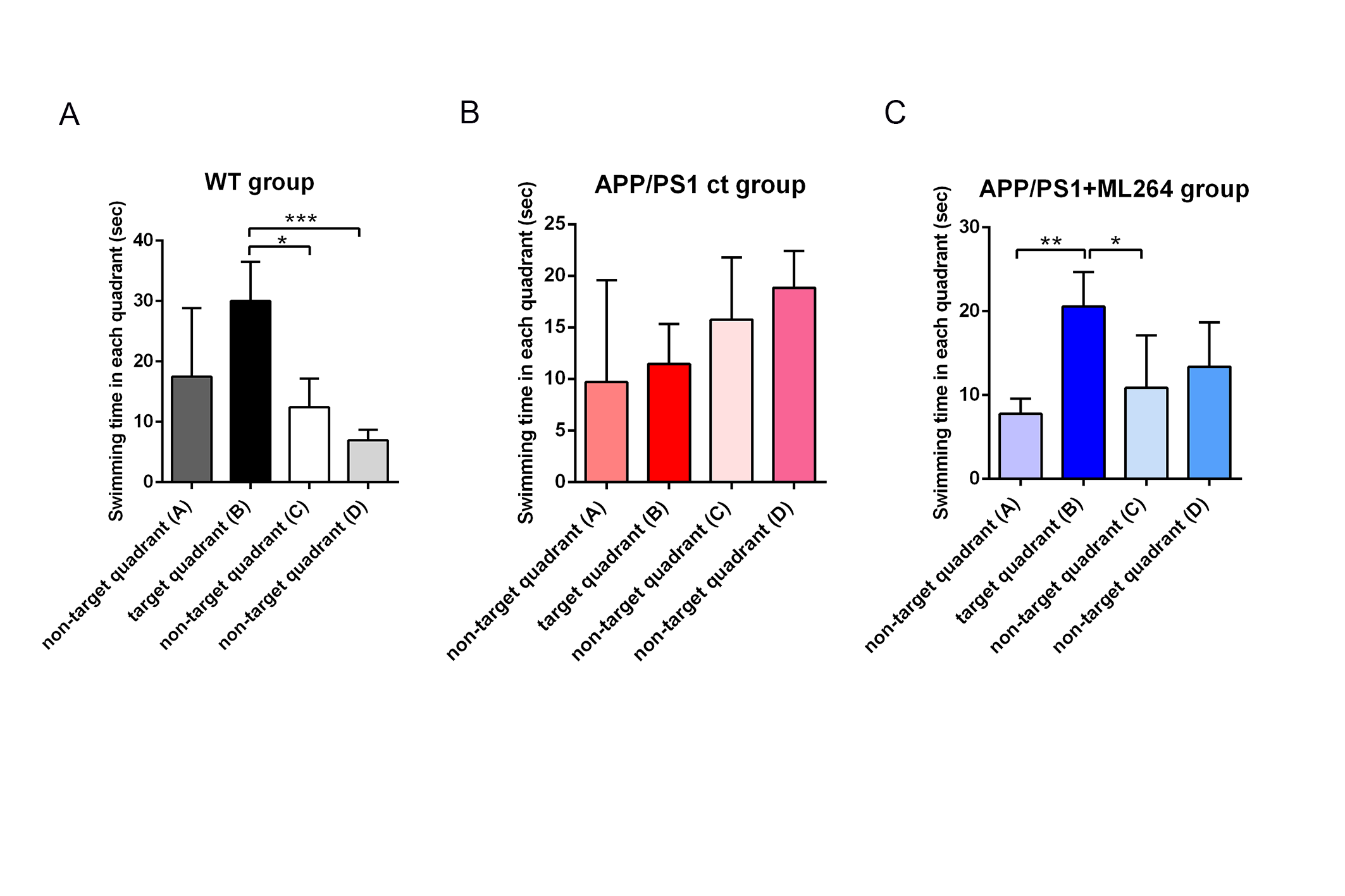

Supplement: Supplementary file 3 — Additional file 3: Supplementary Figure S3. The swimming time in each quadrant of the different groups of mice. A The swimming time in each quadrant of WT mice (n = 6). B The swimming time in each quadrant of vehicle-treated APP/PS1 mice (n = 6). C The swimming time in each quadrant of ML264-treated APP/PS1 mice (n = 6). Data in A-C are mean ± s.e.m (*P < 0.05, **P < 0.01, and ***P < 0.001; one-way ANOVA). [file 13195_2022_1050_MOESM3_ESM.tif]
