# Supplementary material for: Krüppel-like factor 5 accelerates the pathogenesis of Alzheimer’s disease via BACE1-mediated APP processing
Source: Alzheimers Res Ther. 2022 Jul 26;14:103. doi: 10.1186/s13195-022-01050-3 (PMC9316766; doi:10.1186/s13195-022-01050-3)
Supplement: Supplementary file 4 — Additional file 4: Supplementary Table S1. Target sequences of short-hairpin RNA (shRNA) targeting KLF5 (KLF5-shRNA) and BACE1 (BACE1-shRNA). [file 13195_2022_1050_MOESM4_ESM.pdf]

**Supplementary Table S1** Target sequences of Short-hairpin RNA (shRNA) targeting KLF5 (KLF5-shRNA) and BACE1 (BACE1-shRNA).

| Target sequences | |
|---|---|
| KLF5-shRNA | 5'-CGTATCCACTTCTGCGATTAT-3' |
| BACE1-shRNA | 5'-CGTCATGATGGAAGGTTTCTA-3' |
