# Supplementary material for: Krüppel-like factor 5 accelerates the pathogenesis of Alzheimer’s disease via BACE1-mediated APP processing
Source: Alzheimers Res Ther. 2022 Jul 26;14:103. doi: 10.1186/s13195-022-01050-3 (PMC9316766; doi:10.1186/s13195-022-01050-3)
Supplement: Supplementary file 5 — Additional file 5: Supplementary Table S2. Primer sequences for real-time PCR and ChIP-qPCR. [file 13195_2022_1050_MOESM5_ESM.pdf]

Supplementary Table S2 Primer sequences for real-time PCR and ChIP-qPCR.

Primers for real-time PCR	
Gene	Primer Sequences
mKLF5	F: 5’-CCGGAGACGATCTGAAACACG-3’
	R: 5’-GTTGATGCTGTAAGGTATGCCT-3’
mBACE1	F: 5’-ACATATCGAGACCTCCGAAAGG-3’
	R: 5’-AACTTGTCCGATTCAGTGATGG-3’
m β-actin	F: 5’-GGTCAGAAGGACTCCTATGTGG-3’
	R: 5’-TGTCGTCCCAGTTGGTAACA-3’
hKLF5	F: 5’-CCTGGTCCAGACAAGATGTGA-3’
	R: 5’-GAACTGGTCTACGACTGAGGC-3’
hBACE1	F: 5’-ACCAACCTTCGTTTGCCCAA-3’
	R: 5’-TCTCCTAGCCAGAAACCATCAG-3’
hGAPDH	F: 5’-GGTCGGAGTCAACGGATTTG-3’
	R: 5’-GGAAGATGGTGATGGGATTTC-3’
Primers for ChIP-qPCR	
hBACE1 promoter Site4	F: 5’-GGCATGAGAACTGCTTGA-3’
	R: 5’-AGGCATGAGAACTGCTTGA-3’
mBACE1 promoter Site1 / Site2	F: 5’-ATGAGGGCATTGGCAGAA-3’
	R: 5’-CTTACAGGAGCGAGACCC-3’
mBACE1 promoter Site3	F: 5’-CTCTACCTCCTAAGTGCTGG-3’
	R:5’-GATTAACCGGGGAGAACTG-3’
