# Supplementary material for: Krüppel-like factor 5 accelerates the pathogenesis of Alzheimer’s disease via BACE1-mediated APP processing
Source: Alzheimers Res Ther. 2022 Jul 26;14:103. doi: 10.1186/s13195-022-01050-3 (PMC9316766; doi:10.1186/s13195-022-01050-3)
Supplement: Supplementary file 6 — Additional file 6: Supplementary Table S3. Characterization of patients in different CSF groups. MCI: Mild cognitive impairment; DAT: Alzheimer’s type of dementia; MMSE: Mini-Mental State Examination; and MoCA: Montreal Cognitive Assessment (****P < 0.0001; data versus MCI; Student’s test). MCI: Mild cognitive impairment; DAT: Alzheimer’s type of dementia. ****P<0.0001, the data were analyzed by Student’s test, vs. MCI. [file 13195_2022_1050_MOESM6_ESM.pdf]

Supplementary Table S3 Characterization of patients in different CSF groups.

Characteristics	Normal cognition (n=30)	MCI (n=30)	DAT(n=30)
**Gender (n)**			
Male	15	14	12
Female	15	16	18
**Age**			
Mean ± SEM	60.07 ±1.249	63.43 ±1.080	60.47 ±1.297
**MMSE score**			
Mean ± SEM	-	25.32±0.5611	13.63±1.099****
**MoCA score**			
Mean ± SEM	-	19.80±0.7942	8.833±0.8658****

MCI: Mild cognitive impairment; DAT: Alzheimer's type of dementia; MMSE: Mini-mental State Examination; MoCA: Montreal Cognitive Assessment.

****P<0.0001, the data were analyzed by Student's test, vs. MCI.
