# Supplementary material for: Krüppel-like factor 5 accelerates the pathogenesis of Alzheimer’s disease via BACE1-mediated APP processing
Source: Alzheimers Res Ther. 2022 Jul 26;14:103. doi: 10.1186/s13195-022-01050-3 (PMC9316766; doi:10.1186/s13195-022-01050-3)
Supplement: Supplementary file 7 — Additional file 7: Supplementary Table S4. Characterization of patients in different serum groups. HC: Healthy control; MCI: Mild cognitive impairment; and DAT: Alzheimer’s type of dementia (*P < 0.05; data versus MCI; Student’s test). HC: Healthy control; MCI: Mild cognitive impairment; DAT: Alzheimer’s type of dementia. *P<0.05, the data were analyzed by Student’s test, vs. MCI. [file 13195_2022_1050_MOESM7_ESM.pdf]

Supplementary Table S4 Characterization of patients in different serum groups.

Characteristics	HC (n=40)	MCI (n=30)	DAT(n=30)
**Gender (n)**			
Male	20	16	12
Female	20	14	18
**Age**			
Mean ± SEM	69.95 ±1.456	68.77 ±1.805	73.17 ±1.626
**MMSE score**			
Mean ± SEM	-	18.50±2.262	13.82±2.554
**MoCA score**			
Mean ± SEM	-	15.79±1.403	10.36±2.073*

HC: Healthy control; MCI: Mild cognitive impairment; DAT: Alzheimer's type of dementia.

*P<0.05, the data were analyzed by Student's test, vs. MCI.
